# Effects of first-line chemotherapy on natural killer cells in adult T-cell leukemia–lymphoma and peripheral T-cell lymphoma

**DOI:** 10.1007/s00280-016-3070-2

**Published:** 2016-06-11

**Authors:** Michinori Ogura, Takashi Ishida, Kunihiro Tsukasaki, Takeshi Takahashi, Atae Utsunomiya

**Affiliations:** Department of Hematology, Tokai Central Hospital, Kakamigahara, Gifu 504-8601 Japan; Department of Hematology and Oncology, Nagoya Daini Red Cross Hospital, Nagoya, Aichi 466-8650 Japan; Department of Hematology and Oncology, Nagoya City University Graduate School of Medical Sciences, Nagoya, Aichi 467-8601 Japan; Department of Hematology, National Cancer Center Hospital East, Kashiwa, Chiba 277-8577 Japan; Department of Hematology and Molecular Medicine, Nagasaki University Graduate School of Biomedical Science, Nagasaki-shi, Nagasaki 852-8523 Japan; Oncology R&D Unit, R&D Division, Kyowa Hakko Kirin Co., Ltd., Chiyoda-ku, Tokyo 100-8185 Japan; Department of Hematology, Imamura Bun-in Hospital, Kamoikeshinmachi, Kagoshima, 890-0064 Japan

**Keywords:** Antibody, ATL, Chemotherapy, NK cell, PTCL

## Abstract

**Purpose:**

Natural killer (NK) cells are well known to be the most important effector cells mediating antibody-dependent cellular cytotoxicity (ADCC) which is an important mechanism of action of antibody drugs. We evaluated the effects of chemotherapy on the cell number and activity of NK cells from patients who received the vincristine–cyclophosphamide–doxorubicin–prednisone (VCAP), doxorubicin–ranimustine–prednisone (AMP), and vindesine–etoposide–carboplatin–prednisone (VECP) (mLSG15) or mLSG15-like (-L) regimen, which is one of the standard of cares for newly diagnosed adult T-cell leukemia–lymphoma (ATL), or the cyclophosphamide–doxorubicin–vincristine–prednisone (CHOP) or CHOP-L regimen which is another standard of care for ATL and peripheral T-cell lymphoma (PTCL).

**Methods:**

The number of lymphocytes and NK cells, and NK cell activity, were assessed using flow cytometry and a ^51^Cr release assay, respectively.

**Results:**

A total of 26 patients with untreated ATL or PTCL were enrolled, and blood samples from 25 patients were evaluable. NK cell number in ATL decreased after mLSG15/-L treatment, and the degree of decrease in the NK cell number was more prominent just before VECP therapy (Day 15–17 of each cycle) than just before VCAP therapy (Day 1 of each cycle). The NK cell number in ATL after CHOP/-L treatment also decreased. Interestingly, the NK cell activity showed a tendency to increase after the treatment. NK cell number in PTCL did not decrease by CHOP/-L regimen, but the activity was slightly decreased after the treatment.

**Conclusions:**

These results indicate that the effects of chemotherapeutic agents on NK cells vary according to the disease type and intensity of chemotherapy.

## Introduction

ATL belongs to the category of mature T/NK-cell neoplasms [1] and occurs secondary to HTLV-1 infection [2–4]. ATL frequently occurs in the HTLV-1 endemic area in Kyushu islands, the western part of Japan [5], and its prognosis is the worst among common subtypes of mature T/NK-cell neoplasms [6]. Recently, VCAP–AMP–VECP regimen (mLSG15), which was a modified version of LSG15 [7], developed by the Japan Clinical Oncology Group (JCOG) has been established as a standard of care in newly diagnosed aggressive ATL [8]. However, the results are still not satisfactory, with a median survival of 13 months and a 3-year overall survival (OS) rate of only 24 % [9]. Other mature T/NK-cell neoplasms include peripheral T-cell lymphoma, not otherwise specified (PTCL-NOS), angioimmunoblastic T-cell lymphoma (AITL), and anaplastic large cell lymphoma (ALCL). These disease subtypes are collectively called PTCL [1]. The current standard of care for PTCL is CHOP therapy including the CHOP-L regimen [10]; however, it has been reported that PTCL shows a generally poor prognosis, with 5-year OS rates of only 32 % in PTCL-NOS and AITL, except for in anaplastic lymphoma kinase (ALK)-positive ALCL, which is associated with a 5-year OS rate of 70 % [6].

In recent years, the efficacies of various therapeutic monoclonal antibody (mAb)-based monotherapies have been investigated in PTCL. These mAbs include the anti-CD52 mAb alemtuzumab [11, 12], the anti-CC chemokine receptor 4 (CCR4) mAb mogamulizumab [13–15], and the anti-CD30 antibody drug conjugate mAb, brentuximab vedotin [16]. Based on evidence indicating that a combination of the anti-CD20 mAb rituximab [17], and chemotherapy including CHOP therapy achieved a marked improvement in therapeutic efficacy in patients with B-cell non-Hodgkin lymphomas, the combination of mAbs and chemotherapy is expected to represent a promising regimen for mature T/NK-cell neoplasms.

ADCC is an important tumor cell-killing mechanism of action of mAbs including the anti-CCR4 mAb mogamulizumab, which has been approved for CCR4-positive ATL and relapsed/refractory PTCL in Japan. Mogamulizumab is a defucosylated mAb, resulting in highly enhanced ADCC by increased binding affinity to the Fcγ III receptor (CD16) on effector cells [18–20]. It has been also shown that the ADCC of mogamulizumab mainly depends on the amount of effector cells such as CD16/CD56 double-positive NK cells [19]; however, to our knowledge, there have been no reports regarding the effects of various chemotherapeutic agents on CD16/CD56 double-positive NK cells cause high ADCC or other effector cells to date. In this regard, we carried out the present study to evaluate the effects of two first-line therapies, namely the mLSG15 or mLSG15-L (mLSG15/-L) and CHOP or CHOP-L (CHOP/-L) regimens, on NK cell number and activity in newly diagnosed ATL and PTCL patients.

## Materials and methods

### Study design

This study was carried out in four institutions (Nagoya Daini Red Cross Hospital, Nagoya City University Hospital, Nagasaki University Hospital, and Imamura Bun-in Hospital) in collaboration with Kyowa Hakko Kirin Co., Ltd. (Tokyo, Japan) from May 2009 to November 2010. As per the study protocol, blood samples were collected at specified time points from patients with newly diagnosed ATL or PTCL receiving the mLSG15/-L regimen or CHOP/-L regimen as the primary treatment. Cell numbers of lymphocytes and NK cells, and NK cell activity, were determined by flow cytometry, and ^51^Cr release assay, respectively [21]. Blood samples were obtained at eight time points before the VCAP and VECP therapies in the first to the fourth cycle of the mLSG15/-L regimen, and at eight points before the first to the eighth cycle of the CHOP/-L regimen. A blood sample of 10 mL was obtained at each time point, and the measurement was terminated when the treatment was discontinued or changed to another treatment.

Blood samples were also collected from healthy adult volunteers in a clinical research institution between November and December 2009, and their NK cell number and activity were measured in the same manner.

### Patients and healthy adult volunteers

Patients with newly diagnosed and previously untreated ATL (acute, lymphoma, or unfavorable chronic type) or PTCL aged 20 years or older to whom it was planned to administer the mLSG15/-L or CHOP/-L regimen were eligible for the study. The unfavorable chronic type of ATL was defined by the presence of at least one of the following three factors: low serum albumin, high lactate dehydrogenase, or high blood urea nitrogen concentration [9].

The healthy adult volunteers were aged 40 or above who were negative for hepatitis B virus surface antigen, hepatitis C virus antibody, human immunodeficiency virus antibody, or syphilis test such as rapid plasma regain and treponema pallidum hemagglutination test. The blood test for HTLV-1 antibodies was not carried out for healthy adult volunteers. They had also no past history of allergic diseases such as atopic dermatitis, bronchial asthma, or hay fever. All patients and healthy adult volunteers signed written informed consent.

### Evaluation criteria and endpoint

The immune status was evaluated in all eligible patients, and variations in NK cell number and activity before, during, and after chemotherapy were determined in order to speculate as to the usefulness of subsequent mAb therapy for these diseases.

The NK cell number was obtained through measurement by flow cytometry (3-color assay for CD45/CD16/CD56 in CD3-negative fraction). Figure 4 in “Appendix” shows the representative FACS plot defined by CD16 and CD56. The lymphocyte gate was determined by side scatter-height (SSC-H) and CD45. The gated cells were displayed on a plot of CD16 versus CD56 expression. The double-positive fraction of CD16/CD56 was defined as the NK cell. The NK cell number was calculated according to the following formula:

$$\begin{aligned} {\text{NK}}\;{\text{cell}}\;{\text{number}}\;\left( {/\upmu{\text{L}}} \right) & = {\text{lymphocyte}}\;{\text{count}}\;\left( {/\upmu{\text{L}}} \right) \\ & \quad \times {\text{double-positive fraction of CD16/CD56}}\;\left( \% \right) \times 0.01.\end{aligned}$$

NK cell activity was obtained through measurement by ^51^Cr release assay (an assay using the reaction of peripheral blood mononuclear cells (monocyte and lymphocyte) and ^51^Cr-labeled K562 cells at an effector/target ratio of 20:1), and was calculated according to the following formula:

$$\begin{aligned} {\text{NK}}\;{\text{cell}}\;{\text{activity}}\;\left( \% \right) & = {\text{Specific}}\;{\text{lysis}}\;\left( \% \right)^{*} \times {\text{double-positive fraction of CD16/CD56}}\;\left( \% \right) \\ & \quad \times 0.01 \\ \end{aligned}$$

^*^(E - S)/(M - S) × 100, where *E* is the experimental release, *S* is the spontaneous release, and *M* is the maximum release.

### Statistical analysis

**Fig. 1:**
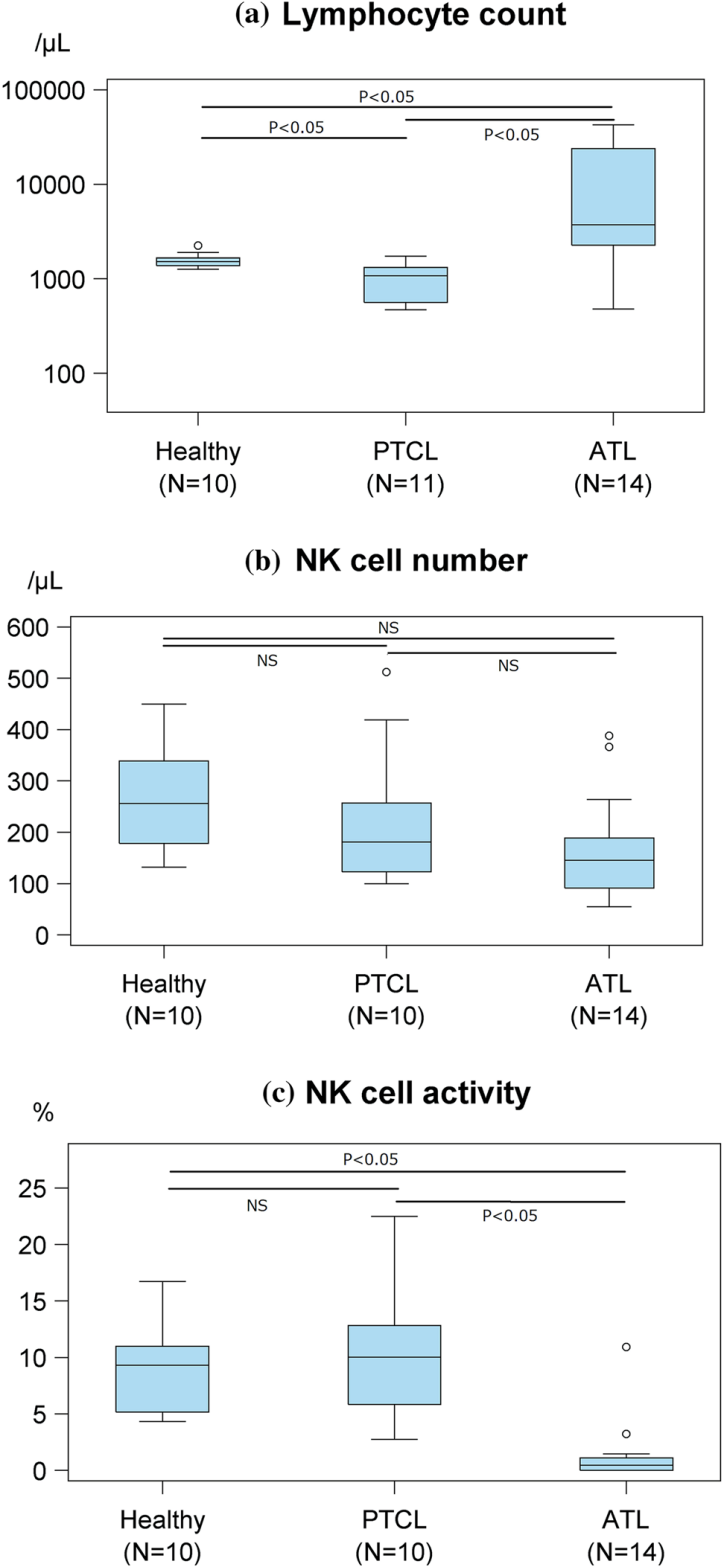
Lymphocyte count, natural killer (NK) cell number, and NK cell activity before treatment initiation as determined using flow cytometry (cell number) and a ^51^Cr release assay (activity). **a** The mean lymphocyte count in healthy volunteers, peripheral T-cell lymphoma (PTCL) patients, and adult T-cell leukemia–lymphoma (ATL) patients were 1580, 991, and 11,618/μL, respectively. **b** The NK cell number in healthy volunteers, PTCL patients, and ATL patients, the corresponding mean values were 262, 224, and 166/μL, respectively. **c** The NK cell activity values in healthy volunteers, PTCL patients, and ATL patients, the corresponding mean values were 8.8, 10.4, and 1.4 %, respectively

Data were shown as box plots. For multiple comparison, Dwass, Steel, Critchlow–Fligner multiple comparison analysis was used as shown in Fig. 1. All statistical analyses were conducted by SAS ver 9.4 (SAS Institute Inc., Cary, NC, USA).

### Study oversight

The study was sponsored by Kyowa Hakko Kirin Co., Ltd. The academic investigators and the sponsor were jointly responsible for the study design. The protocol was approved by the institutional review boards at each participating site, and the study was conducted complying with the ethical guidelines on clinical research and in accordance with the Declaration of Helsinki 1995. The blood sample assays using flow cytometry and ^51^Cr release were outsourced to SRL Medisearch Inc. Data analysis was outsourced to Biostatistics center, Kurume university.

## Results

### Patient characteristics

The total number of patients enrolled was 26, and 25 patients (14 patients with ATL and 11 patients with PTCL) were included in the data analysis. One patient was excluded from analysis due to a low initial lymphocyte count of 80/μL. Data from this patient were rejected because it was judged to be inappropriate to use this value as the basis for examination of variations, and calculation of the NK cell number and activity. Table 1 shows the demographics and clinical characteristics of the 25 analyzed patients, and Table 2 shows the breakdown of patients on chemotherapy in relation to the disease subtype. The mLSG15/-L regimen was administered to 9 (64 %) patients with ATL. It should be noted that although the number of patients analyzed was limited, no marked difference was found in disease subtype according to the type of chemotherapy (mLSG15/-L vs. CHOP/-L). The CHOP/-L regimen was administered to all (100 %) patients with PTCL.

**Table 1:** Patient demographics and clinical characteristics

Characteristic		ATL patients (*n* = 14)		PTCL patients (*n* = 11)	
		*n* (%)		*n* (%)	
Age (years)	Median	56		69	
	Range	47–72		36–78	
Sex	Male	9 (64)		9 (82)	
	Female	5 (36)		2 (18)	
ECOG PS	0	2 (14)		5 (45)	
	1	9 (64)		4 (36)	
	2	2 (14)		2 (18)	
	3	0 (0)		0 (0)	
	4	1 (7)		0 (0)	
Therapy	CHOP/-L	5 (36)		11 (100)	
	mLSG15/-L	9 (64)		0 (0)	
Subtype		Acute	10 (71)	AITL	5 (45)
		Lymphoma	1 (7)	PTCL-NOS	3 (27)
		Chronic	3 (21)	ALCL ALK-	1 (9)
				EATL	1 (9)
				SPTCL	1 (9)

*AITL* angioimmunoblastic T-cell lymphoma, *ALCL* anaplastic large cell lymphoma, *ALK* anaplastic lymphoma kinase, *ATL* adult T-cell leukemia–lymphoma, *CHOP* cyclophosphamide, doxorubicin, vincristine, and prednisone, *CHOP/-L* CHOP or CHOP-like regimen, *EATL* enteropathy-associated T-cell lymphoma, *ECOG* Eastern Cooperative Oncology Group, *mLSG15* vincristine, cyclophosphamide, doxorubicin, prednisone and doxorubicin, ranimustine, prednisone and vindesine, etoposide, carboplatin, prednisone, *mLSG15/-L* mLSG15 or mLSG15-like regimen, *NOS* not otherwise specified, *PS* performance status, *PTCL* peripheral T-cell lymphoma, *SPTCL* subcutaneous panniculitis-like T-cell lymphoma

**Table 2:** Breakdown of patients on chemotherapy in relation to the disease subtype

ATL subtype (*n* = 14)	*n* (%)	Therapy	
		mLSG15/-L *n* (%)	CHOP/-L^a^ *n* (%)
Acute	10 (71)	6 (43)	4 (29)
Lymphoma	1 (7)	1 (7)	0 (0)
Chronic	3 (21)	2 (14)	1 (7)

PTCL subtype (*n* = 11)	*n* (%)	Therapy	
		CHOP *n* (%)	CHOP-L^a^ *n* (%)
AITL	5 (45)	1 (9)	4 (36)
PTCL-NOS	3 (27)	1 (9)	2 (18)
ALCL ALK-	1 (9)	1 (9)	0 (0)
EATL	1 (9)	0 (0)	1 (9)
SPTCL	1 (9)	0 (0)	1 (9)

*AITL* angioimmunoblastic T-cell lymphoma, *ALCL* anaplastic large cell lymphoma, *ALK* anaplastic lymphoma kinase, *ATL* adult T-cell leukemia–lymphoma, *CHOP* cyclophosphamide, doxorubicin, vincristine, and prednisone; *CHOP-L* CHOP-like regimen, *CHOP/-L* CHOP or CHOP-like regimen, *EATL* enteropathy-associated T-cell lymphoma, *mLSG15* vincristine, cyclophosphamide, doxorubicin, prednisone and doxorubicin, ranimustine, prednisone and vindesine, etoposide, carboplatin, prednisone, *mLSG15/-L* mLSG15 or mLSG15-like regimen, *NOS* not otherwise specified, *PTCL* peripheral T-cell lymphoma, *SPTC*L subcutaneous panniculitis-like T-cell lymphoma

^a^THP-COP, vincristine, pirarubicin, cyclophosphamide, and prednisone

Table 3 in “Appendix” shows the breakdown of ATL patients received with mLSG15/-L regimen and CHOP/-L regimen, and Table 4 in “Appendix” shows the breakdown of PTCL patients received with CHOP/-L regimen. Disease progressions were almost reasons for taken off these therapies. None of ATL patients received both mLSG15/-L and CHOP/-L regimens.

**Table 3:** Breakdown of ATL patients received with (a) VCAP (Day 1 of each cycle) and VECP (Day 15–17 of each cycle: ※) of mLSG15/-L regimen, (b) CHOP/-L regimen

	Cycle number							
	1	1※	2	2※	3	3※	4	4※
(a)								
Number of received patients	9	8	8	8	7	6	5	2
Number of analyzed patients	9	8	8	8	7	6	5	2
Number of missing data	–	1	0	0	0	0	0	0
Number of taken off therapy	–	0	1	1	2	3	4	7

	Cycle number							
	1	2	3	4	5	6	7	8
(b)								
Number of received patients	5	5	3	2	2	0	1	1
Number of analyzed patients	5	5	3	2	2	0	1	1
Number of missing data	–	0	0	0	0	2	0	0
Number of taken off therapy	–	0	2	3	3	3	4	4

**Table 4:** Breakdown of PTCL patients received with CHOP/-L regimen

	Cycle number							
	1	2	3	4	5	6	7	8
Number of received patients	11	11	8	9	7	7	6	6
Number of analyzed patients	10	10	7	9	7	7	6	6
Number of missing data	–	0	2	0	1	1	1	0
Number of taken off therapy	–	0	1	1	2	2	3	4

### Lymphocyte counts and NK cell number and activity before treatment initiation

Figure 1 shows the lymphocyte count, NK cell number, and NK cell activity determined in 14 patients with ATL, 11 patients with PTCL, and 10 healthy adult volunteers. The lymphocyte count before initiation of treatment was significantly higher by 1 log in ATL compared to in PTCL patients (Fig. 1a), which was probably attributable to the fact that patients with acute-type ATL who had ATL cells in the peripheral blood [22] accounted for 71 % of the patients with ATL (Table 2). There was no significant difference in the number of CD16/CD56 double-positive NK cells which can contribute to ADCC of mogamulizumab, before treatment initiation between ATL and PTCL patients, or between these patients and healthy adult volunteers (Fig. 1b). Although the NK cell activity before treatment initiation was markedly lower in ATL than in PTCL patients (Fig. 1c), there was no significant difference in the NK cell activity between patients with PTCL and healthy adult volunteers (Fig. 1c).

### Variations in the NK cell number and activity in ATL patients after treatment initiation

**Fig. 2:**
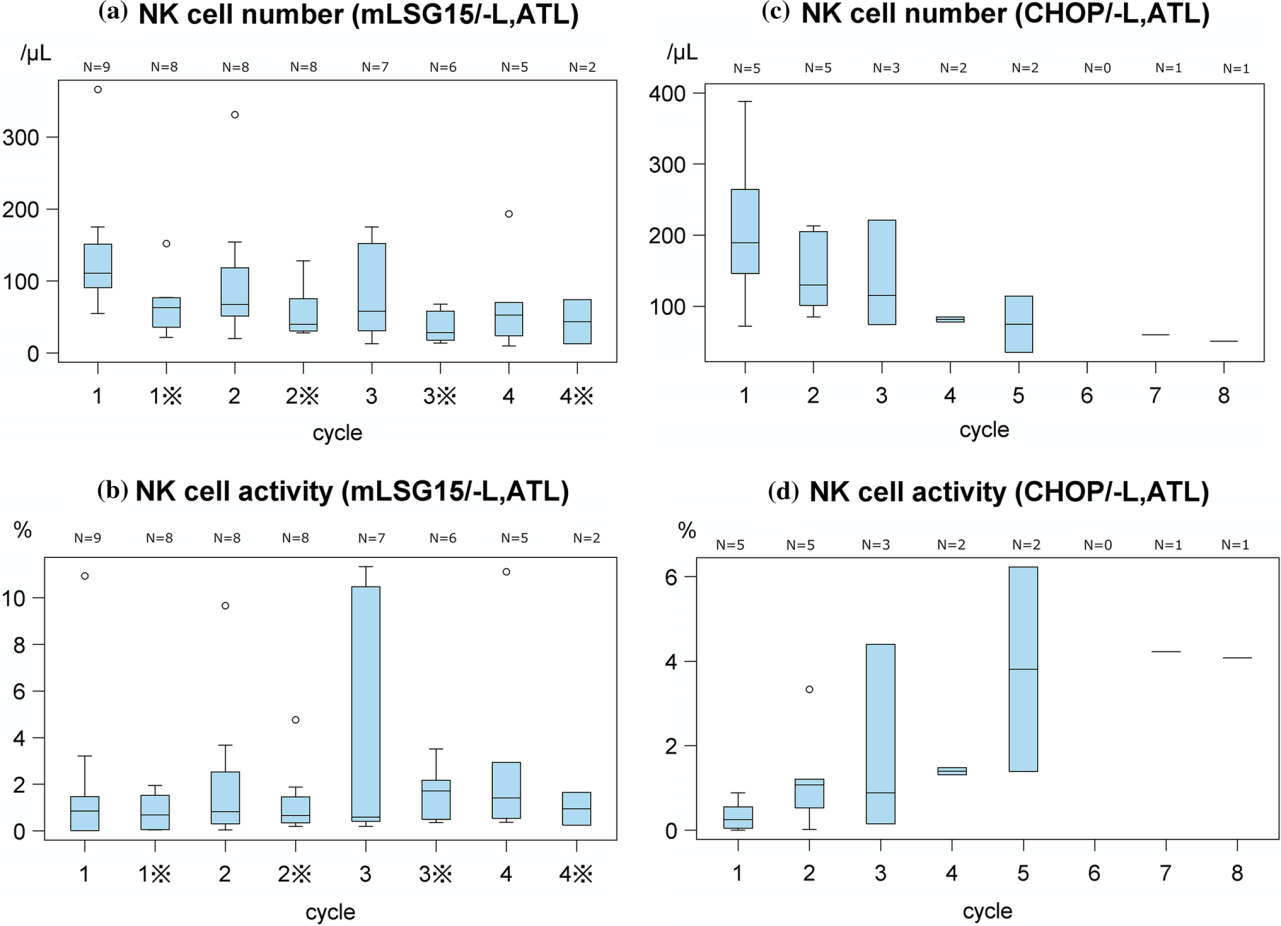
Variations in natural killer (NK) cell number and NK cell activity in adult T-cell leukemia–lymphoma (ATL) patients. **a** NK cell number determined before (Pre) administration at VCAP (Day 1 of each cycle) and VECP (Day 15–17 of each cycle: ※) of mLSG15/-L treatment initiation. **b** NK cell activity determined before (Pre) administration at VCAP (Day 1 of each cycle) and VECP (Day 15–17 of each cycle: ※) of mLSG15/-L treatment initiation. **c** NK cell number determined before (Pre) administration at each cycle of CHOP/-L treatment initiation. **d** NK cell activity determined before (Pre) administration at each cycle of CHOP/-L treatment initiation

The NK cell number in patients with ATL was decreased as a result of the mLSG15/-L regimen, and this decrease was more prominent before VECP therapy (Day 15–17 of each cycle) than before VCAP therapy (Day 1 of each cycle) (Fig. 2a). The NK cell number in patients with ATL also decreased by the CHOP/-L regimen (Fig. 2c). However, the NK cell activity by the CHOP/-L regimen showed a tendency to increase (Fig. 2d), although there was not marked difference by mLSG15/-L regimen (Fig. 2b).

### Variations in NK cell number and activity in PTCL patients after treatment initiation

**Fig. 3:**
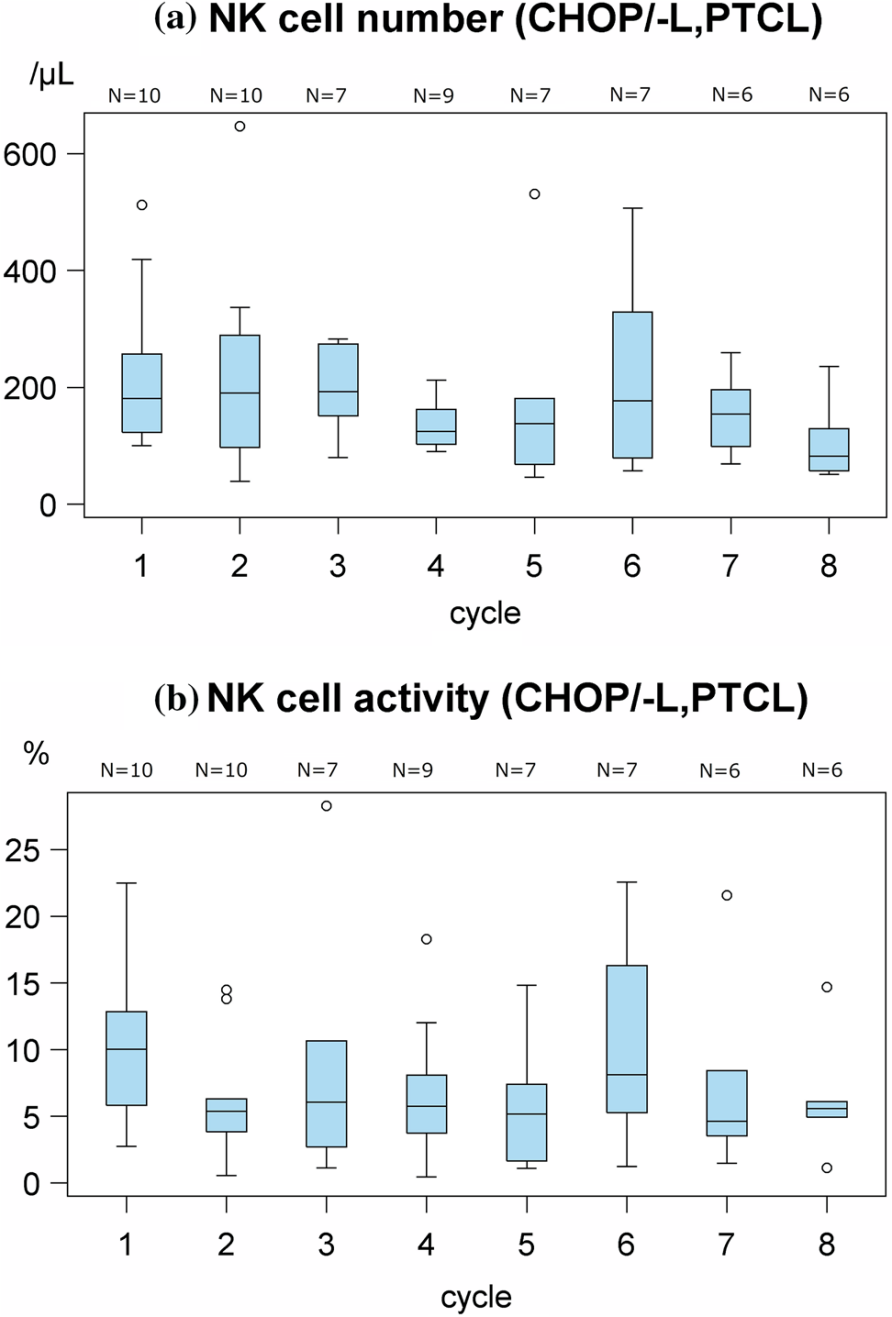
Variations in natural killer (NK) cell number and NK cell activity in peripheral T-cell lymphoma (PTCL) patients. **a** NK cell number determined before (Pre) administration at each cycle of CHOP/-L treatment initiation. **b** NK cell activity determined before (Pre) administration at each cycle of CHOP/-L treatment initiation

**Fig. 4:**
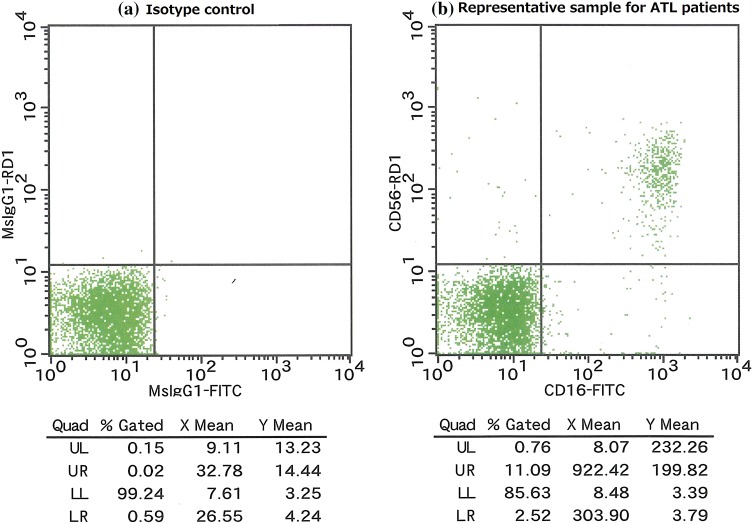
Representative FACS plot defined by CD16 and CD56. **a** Isotype control and **b** representative sample for ATL patients

In PTCL patients, the NK cell number did not decrease by the CHOP/-L regimen (Fig. 3a), though the NK cell number in patients with ATL decreased by this regimen (Fig. 2c). The NK cell activity in PTCL patients slightly decreased by the CHOP/-L regimen (Fig. 3b).

## Discussion

In this study, the effects of first-line therapy for newly diagnosed ATL and PTCL patients on NK cells defined as CD16/CD56 double-positive ones can cause high ADCC for defucosylated antibodies such as mogamulizumab were evaluated using NK cell number and NK cell activity substitute functional cell vitality as indices. These data showed that the effects of chemotherapy on NK cells varied according to the disease (ATL or PTCL) and the regimen used and/or the number of courses of chemotherapy (mLSG15/-L regimen or CHOP/-L regimen). Furthermore, it was also found that the NK cell activity before treatment initiation was markedly lower in ATL than in PTCL patients. This low activity of NK cell in ATL may be caused by suppression of NK cells by a subset of ATL cells functioning as Treg cells [23–25]. These results suggest that it is more difficult for some therapeutic mAbs that require an effector function to exert their therapeutic effects in ATL compared to in PTCL, although the mechanism of NK cell activity may be different from that of ADCC. However, it cannot be directly concluded that therapeutic Abs are less likely to exert benefits in patients with ATL. In fact, the NK cell number in cases treated with the mLSG15/-L regimen tended to be restored before the initiation of each chemotherapeutic cycle (VCAP therapy) in comparison with before toward the end of each cycle (VECP therapy). This finding suggests that in cases where mAbs are combined with the mLSG15/-L regimen, some therapeutic mAbs may be more likely to exert their beneficial effects when they are used before the initiation of each cycle. Additionally, the NK cell activity tended to increase after the CHOP/-L regimen than after the mLSG15/-L regimen in patients with previously untreated ATL. Therapeutic effect of the administered chemotherapy against more suppressive ATL cells compared to PTCL may result in increased vitality per cell of survived NK cells in ATL patients. However, the details of why CHOP/-L treatment tended to produce better results compared to those of more potent intensive regimen, mLSG15/-L, are necessary to carefully consider, while paying heed to direct effects on NK cells by each chemotherapy itself. Therefore, it is not possible to determine which of the two regimens, both of which consist of drugs with different solitary effects, should be recommended as the most suitable chemotherapy to be combined with therapeutic Abs. However, the CHOP/-L regimen, which was associated with a less prominent decrease in totally NK cell activity, seems to be more promising, assuming that patients respond similarly to chemotherapy alone. Further investigations in a greater number of patients are needed, because this was a small-scale study.

Recently, it was carried out a phase II controlled clinical study (clinical trial information: NCT01173887) to examine the effects of combination treatment using the mLSG15 regimen and mogamulizumab in patients with untreated ATL [26]. In that phase II study, mogamulizumab was administered at intervals of 2 weeks (at the same time as VCAP and VECP administration) over four courses of the mLSG15 regimen (8 times in total), i.e., at the same times as the blood sampling was performed in the present study. The results showed that the complete response rate and overall response rate in the 29 patients administered mLSG15 + mogamulizumab versus the 24 patients administered the mLSG15 alone were 52 versus 33 % and 86 versus 75 %, respectively, with mLSG15 combined with mogamulizumab showing better efficacy in both indices. These data indicated that combining mogamulizumab with mLSG15 showed an additive effect with mLSG15, which tended to cause a greater decrease in NK cell number than the CHOP/-L regimen. Therefore, we speculate that mogamulizumab combination with the CHOP/-L regimen could also be beneficial for patients in whom the mLSG15 regimen is difficult to implement because of advanced age or for other reasons. Further investigations are needed to examine this presumption.

On the other hand, in patients with PTCL, the decrease in NK cell number and activity after the standard chemotherapy, i.e., the CHOP/-L regimen, was relatively small, as compared to the CHOP/-L treated patients with ATL, who would be immune suppressive condition as seen in Fig. 1c which shows significant lower activity of NK cells in ATL patients compared to PTCL. This result suggests that the effect of combining therapeutic Abs with chemotherapy is considerably more promising in PTCL patients. Indeed, a phase II clinical study of alemtuzumab in combination with CHOP demonstrated favorable effects, with complete response and overall response rates in 24 patients of 71 and 75 %, respectively [27]. The efficacy of mogamulizumab in combination with CHOP in patients with untreated primary PTCL is also expected.

In conclusion, the results of the present study provide an important basis for future investigations of the combined effects of chemotherapy and therapeutic Abs [18–20] whose activity is affected by CD16/CD56 double-positive NK cells. Further analysis of CD16-positive other effector cells which antibodies bind also remains to be explored to fully understand the combination effects.

## Appendix

See Tables 3, 4 and Fig. 4.

## Abbreviations

ADCC: Antibody-dependent cellular cytotoxicity
AITL: Angioimmunoblastic T-cell lymphoma
ALCL: Anaplastic large cell lymphoma
ALK: Anaplastic lymphoma kinase
ATL: Adult T-cell leukemia–lymphoma
CCR4: Chemokine receptor 4
CD: Cluster of differentiation
CHOP: Cyclophosphamide, doxorubicin, vincristine, and prednisone
JCOG: Japan Clinical Oncology Group
mLSG15: VCAP–AMP–VECP; vincristine, cyclophosphamide, doxorubicin, and prednisone; doxorubicin, ranimustine, and prednisone; and vindesine, etoposide, carboplatin, and prednisone
NK: Natural killer
OS: Overall survival
PTCL: Peripheral T-cell lymphoma
PTCL-NOS: Peripheral T-cell lymphoma, not otherwise specified
SSC-H: Side scatter-height
